# Gintonin-Induced Wound-Healing-Related Responses Involve Epidermal-Growth-Factor-like Effects in Keratinocytes

**DOI:** 10.3390/ijms241814094

**Published:** 2023-09-14

**Authors:** Kyung-Jong Won, Rami Lee, Sun-Hye Choi, Ji-Hun Kim, Sung-Hee Hwang, Seung-Yeol Nah

**Affiliations:** 1 Department of Physiology and Medical Science, College of Medicine, Konkuk University, Chungju 27478, Republic of Korea; kjwon@kku.ac.kr; 2 Ginsentology Research Laboratory, Department of Physiology, College of Veterinary Medicine, Konkuk University, Seoul 05029, Republic of Korea; rmlee12@konkuk.ac.kr (R.L.); bioskjh@konkuk.ac.kr (J.-H.K.); 3 Department of Animal Health, College of Health and Medical Services, Osan University, Osan 18119, Republic of Korea; vettman@osan.ac.kr; 4 Department of Pharmaceutical Engineering, College of Health Sciences, Sangji University, Wonju 26339, Republic of Korea

**Keywords:** gintonin, epidermal growth factor receptor, keratinocyte, proliferation, skin, wound healing

## Abstract

Epidermal growth factor (EGF) receptor activation and related downstream signaling pathways are known to be one of the major mechanisms of the proliferation and migration of keratinocytes. The heparin-binding EGF-like growth factor (HB-EGF) binds to EGF receptors and stimulates keratinocyte proliferation and migration. Gintonin, a novel ginseng compound, is a lysophosphatidic acid (LPA) receptor ligand. Gintonin has skin-wound-healing effects. However, the underlying mechanisms for these gintonin actions remain unclear. In this study, we aimed to elucidate the involvement of EGFRs in gintonin-induced wound repair in HaCaT keratinocytes. In this study, a water-soluble tetrazolium salt-based assay, a modified Boyden chamber migration assay, and immunoblotting were performed. Gintonin increased EGF receptor activation in HaCaT cells. However, the gintonin-induced phosphorylation of the EGF receptor was markedly reduced via treatment with the LPA inhibitor Ki16425 or the EGF receptor inhibitor erlotinib. Gintonin-enhanced proliferation and migration were blocked by the EGF receptor inhibitors (erlotinib and AG1478). Additionally, gintonin stimulated the expression and release of HB-EGF in HaCaT cells. EGF receptor inhibitors blocked gintonin-enhanced HB-EGF expression. These results indicate that the wound-healing effects of gintonin are closely related to the collaboration between EGF receptor activation and HB-EGF release-mediated downstream signaling pathways.

## 1. Introduction

The skin is the first-line protective barrier from the environment, so injuries must be repaired quickly. Keratinocytes are the main components of the epidermis and play an important role in maintaining the skin barrier in response to various growth mediators [1,2,3,4]. In particular, for the re-epithelization of the epidermis, the proliferation and migration of keratinocytes are crucial for wound repair. Growth factors that induce keratinocyte proliferation and/or migration include the epidermal growth factor family, insulin family, fibroblast growth factor family, vascular endothelial growth factor family, chemokines, cholinergic receptor agonists, etc. [1,5]. Members of the epidermal growth factor (EGF) family, such as EGF and heparin-binding EGF-like growth factor (HB-EGF), bind to EGF receptors and activate the proliferation and migration of keratinocytes [1,5,6]. Endogenous sources of EGF include fibroblasts, macrophages, and platelets [1,5]. EGF stimulates keratinocyte proliferation and migration via a paracrine mechanism [1,3,6]. The proliferation and migration of keratinocytes, key events in skin wound healing, are induced through the activation of the phosphoinositide 3-kinases (PI3K)/protein kinase B (AKT) and mitogen-activated protein kinase kinases (MAPKs), which are EGF receptor signaling pathways [7,8,9,10]. EGF is found in many different sources, such as urine, tears, saliva, milk, and plasma [1]; however, it is difficult to isolate and purify from these sources, and EGF itself is not sufficiently stable for use in medical or cosmetic applications. To overcome these drawbacks, many researchers have attempted to develop EGF delivery systems [9,11,12,13] and stable molecules that directly or indirectly activate EGF receptors [14,15]. G-protein-coupled receptor (GPCR) ligands, including lysophosphatidic acid (LPA), transactivate the EGF receptor by enhancing the tyrosine phosphorylation of the EGF receptor and stimulating the proliferation of keratinocytes [16,17]. HB-EGF is an autocrine and paracrine keratinocyte growth factor detected in wound fluid in vivo [1,18,19,20]. At the cellular level, HB-EGF binds to the EGF receptor and activates the proliferation and migration of keratinocytes [1,20,21,22,23,24]. These results suggest that HB-EGF plays an important role in skin wound healing.

Recently, we identified gintonin, an LPA receptor ligand derived from ginseng. Gintonin is a glycoprotein that exerts various physiological and pharmacological effects through LPA receptors, such as LPA [25]. Previous studies have demonstrated that gintonin can induce intracellular calcium transients, cell proliferation, and migration in several cell types, such as HUVEC, human hair follicle cells, and dermal fibroblasts [26,27,28]. We found that gintonin enhanced cell proliferation, migration, vascular endothelial growth factor (VEGF) release, and wound closure in in vitro assays using HaCaT human skin keratinocytes, which are commonly used to study skin wound healing-related keratinocyte activities [3,29], and that it promoted in vivo wound healing in a wounded mouse tail [29]. Moreover, gintonin-enhanced cell proliferation, migration, and VEGF release were markedly blocked via treatment with a LPA inhibitor [29]. Furthermore, extracellular signal-regulated kinase (ERK), calcium chelators, phospholipase C (PLC), and VEGF receptor inhibitors attenuate gintonin-enhanced cell proliferation [29]. These findings indicate that gintonin promotes wound healing by activating LPA receptor–VEGF release-mediated downstream signaling pathways [29]. LPA transactivates the EGF receptor [16,17], and HB-EGF binds to the EGF receptor to activate the proliferation and migration of keratinocytes [20,21,22,23,24]. Therefore, the LPA receptor ligand gintonin may transactivate the EGF receptor and still function through other mechanisms, including the involvement of the EGF receptor and HB-EGF in keratinocyte proliferation and migration.

This study investigated whether or not the EGF receptor is involved in the gintonin-induced wound repair-related migration and proliferation of HaCaT keratinocytes. Furthermore, the gintonin-induced expression and release of HB-EGF in HaCaT cells were analyzed, and the possible role of gintonin-induced HB-EGF in keratinocytes is discussed.

## 2. Results

### 2.1. Gintonin-Mediated Phosphorylation of EGF Receptor

**Figure 1:**
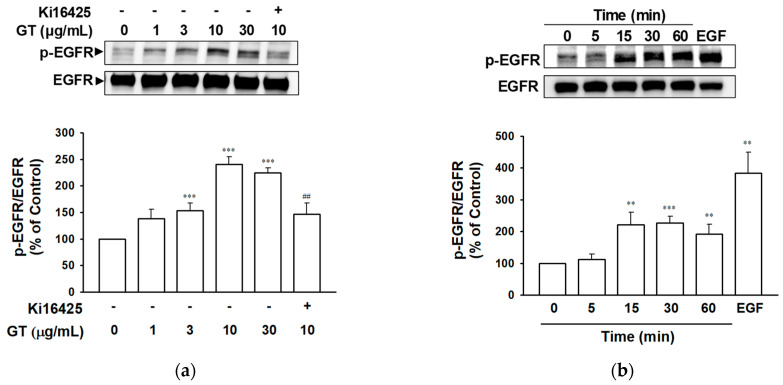
Effect of gintonin on epidermal growth factor receptor phosphorylation in HaCaT cells. (**a**) Cells were serum-starved for 4 h and then incubated with serum-free medium containing gintonin (GT, 1–30 μg/mL) for 30 min in the presence or absence of lysophosphatidic acid (LPA) receptor inhibitor Ki16425 (10 μM). (**b**) Cells were serum-starved for 4 h and then incubated with serum-free medium containing GT (10 μg/mL) for the indicated times or epidermal growth factor (EGF, 4 ng/mL) for 5 min. (**a**,**b**) Cell lysates in each test were immunoblotted using corresponding antibodies. Each upper panel shows the representative images of immunoblotting. Lower statistical graphs represent results expressed as a percentage of untreated control cells. Data are presented as means ± S.E.M. (*n* = 4); ** *p* < 0.01; *** *p* < 0.001 vs. untreated control cells; ^##^ *p* < 0.01 vs. GT 10 μg/mL alone. p-EGFR, phospho-EGF receptor.

To evaluate the effect of gintonin on EGF receptor activation in HaCaT cells, EGF receptor phosphorylation was analyzed via immunoblotting (Figure 1). As shown in Figure 1a, treatment with gintonin in a range of 1–30 μg/mL for 30 min increased the phosphorylation of the EGF receptor in HaCaT cells in a dose-dependent manner, which reached a plateau at 10 μg/mL (Figure 1a). Pretreatment with the LPA1 and LPA3 receptor inhibitor Ki16425 dramatically reduced gintonin-induced EGF receptor phosphorylation, indicating the involvement of the LPA receptor in EGF receptor activation (Figure 1a). Additionally, EGF receptor phosphorylation was observed in HaCaT cells after incubation with gintonin (for 5–60 min). After 5 min of incubation, gintonin (10 μg/mL) did not phosphorylate the EGF receptor, whereas treatment alone with EGF (4 ng/mL) for 5 min strongly phosphorylated the EGF receptor. Gintonin-induced EGF receptor phosphorylation was markedly detected at 15–60 min, showing a delayed response compared to that of EGF.

### 2.2. Gintonin-Induced Phosphorylation of the EGF Receptor and ERK Involves the LPA Receptor as Well as the EGF Receptor

**Figure 2:**
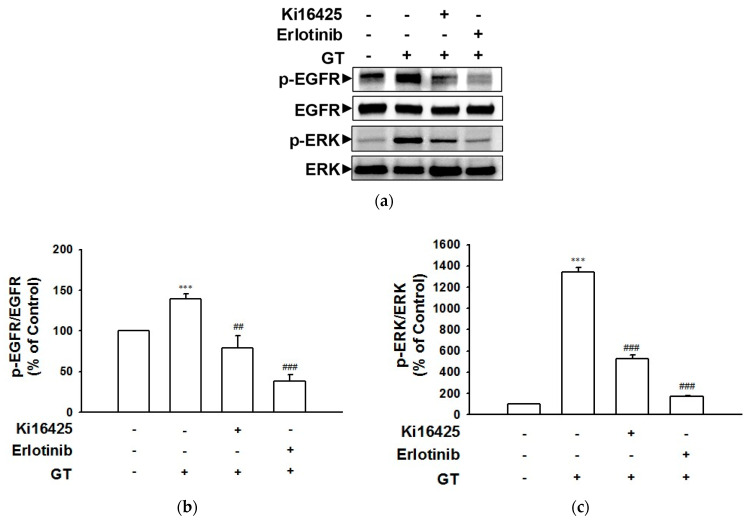
Effects of inhibitors on the gintonin-induced phosphorylation of proteins in HaCaT cells. Cells were serum-starved for 4 h and then incubated with serum-free medium containing gintonin (GT, 10 μg/mL) for 30 min in the presence or absence of lysophosphatidic acid (LPA) receptor inhibitor Ki16425 (10 μM) and epidermal growth factor (EGF) receptor inhibitor erlotinib (0.5 μM). Cell lysates were immunoblotted using corresponding antibodies. (**a**) The representative images of immunoblotting. (**b**,**c**) Statistical graphs based on panel (**a**). Each graph is expressed as a percentage of untreated control cells. Data are presented as means ± S.E.M. (*n* = 4); *** *p* < 0.001 vs. untreated control cells; ^##^ *p* < 0.01; ^###^ *p* < 0.001 vs. GT 10 μg/mL alone. p-EGFR, phospho-EGF receptor; p-ERK, extracellular signal-regulated kinase.

To examine whether or not phosphorylation of the EGF receptor and subsequent ERK are related to LPA and EGF receptor activation, phospho-EGF receptor and phospho-ERK levels in HaCaT cells in the presence and absence of receptor inhibitors were detected via immunoblotting. Ki16425 and erlotinib were used as the LPA1/3 and EGF receptor inhibitors, respectively. As shown in Figure 2, treatment with Ki16425 (10 μM) or erlotinib (0.5 μM) significantly attenuated the gintonin-induced phosphorylation of the EGF receptor (Figure 2a,b) and ERK (Figure 2a,c) in HaCaT cells. These results indicate that gintonin may induce the phosphorylation of the EGF receptor and ERK via the activation of the LPA1/3 receptor subtype and the EGF receptor. The EGF receptor inhibitor erlotinib appears to be a more potent inhibitor than does the LPA1/3 receptor inhibitor Ki16425.

### 2.3. Involvement of EGF Receptor Signaling in Gintonin-Mediated Proliferation and Migration of HaCaT Cells

**Figure 3:**
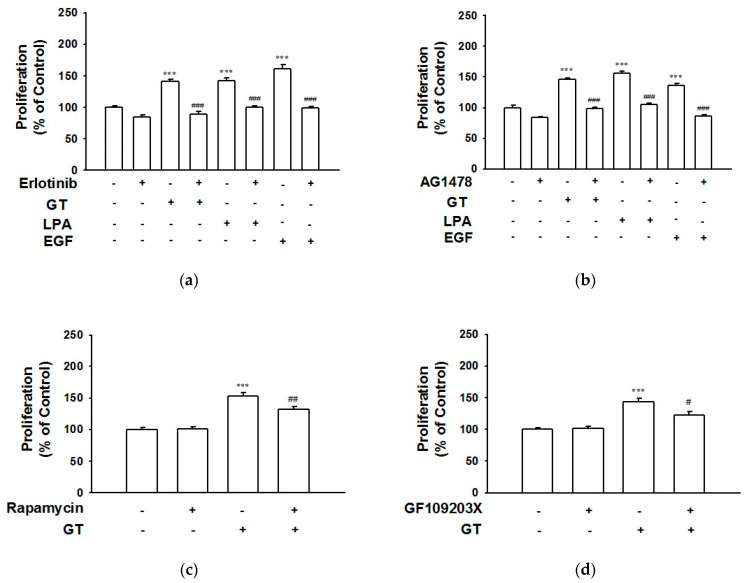
Effect of inhibitors on the proliferation of HaCaT cells. Cells were incubated in serum-free medium with gintonin (GT, 10 μg/mL), lysophosphatidic acid (LPA, 10 μM), or epidermal growth factor (EGF, 4 ng/mL) in the presence or absence of inhibitors for 24 h. Then, a water-soluble tetrazolium formazan (WST)-based assay was performed. Inhibitors used were EGF receptor inhibitor erlotinib (0.5 μM) (**a**), AG1478 (0.5 μM) (**b**), mTOR inhibitor rapamycin (100 nM) (**c**), or protein kinase C inhibitor GF109203X (1 μM) (**d**). LPA and EGF were used as positive controls. The response of untreated cell was considered to be 100%. Data are presented as means ± S.E.M. (*n* = 6); *** *p* < 0.001 vs. untreated control cell; ^#^ *p* < 0.05; ^##^ *p* < 0.01; ^###^ *p* < 0.001 vs. GT, LPA, or EGF alone. mTOR, mammalian target of the rapamycin.

To test whether or not EGF receptor activation participates in the gintonin-induced proliferation and migration of HaCaT cells, we evaluated the in vitro proliferative effect of gintonin using a water-soluble tetrazolium salt (WST)-based assay in HaCaT cells in the presence or absence of EGF receptor inhibitors (Figure 3). Gintonin (10 μg/mL) significantly increased HaCaT cell proliferation. This response was completely blocked via treatment with EGF receptor inhibitors erlotinib (Figure 3a) and AG1478 (Figure 3b), indicating that gintonin-induced proliferation was closely related to EGF receptor activation. EGF receptor inhibitors also reduced LPA (10 μM)- or EGF (4 ng/mL)-induced HaCaT cell proliferation in a manner similar to their effect on gintonin-induced HaCaT cell proliferation.

In addition, pretreatment with the mammalian target of the rapamycin (mTOR) inhibitor rapamycin or the protein kinase C (PKC) inhibitor GF109203X significantly inhibited the gintonin-mediated proliferation of HaCaT cells, demonstrating the involvement of mammalian mTOR and PKC signaling in the gintonin-mediated proliferation of HaCaT cells (Figure 3c,d).

**Figure 4:**
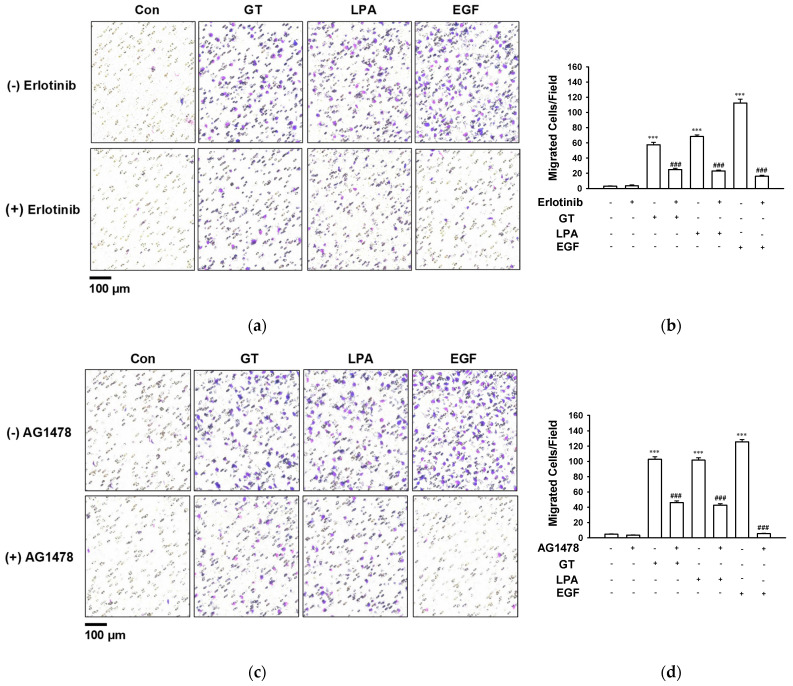
Effect of epidermal growth factor (EGF) receptor inhibitors on the migration of HaCaT cells. (**a**,**c**) Representative images of migrated cells. Cells were pretreated in serum-free medium for 1 h with EGF receptor inhibitor erlotinib (0.5 μM) (**a**) or AG1478 (0.5 μM) (**c**) in the modified Boyden chamber and then cells were incubated with gintonin (GT, 10 μg/mL), lysophosphatidic acid (LPA, 10 μM), or EGF (4 ng/mL) in the presence or absence of inhibitors for 2 h. The migration of cells was analyzed via the modified Boyden chamber assay as described in the Section 4. LPA and EGF were used as positive controls. Migrated cells are shown as violet spots. Scale bar: 100 μm. (**b**,**d**) Statistical graphs obtained from panel (**a**,**c**), respectively. Data represent the means ± S.E.M. (*n* = 16); *** *p* < 0.001 vs. untreated cells (Con); ^###^ *p* < 0.001 vs. GT, LPA, or EGF alone.

The chemotactic migration of HaCaT cells was measured using a modified Boyden chamber (Figure 4). Gintonin induced the migration of HaCaT cells at 10 μg/mL. Gintonin-induced migration was significantly inhibited via treatment with erlotinib (Figure 4a,b) or AG1478 (Figure 4c,d), indicating the involvement of EGF receptor activation in the gintonin-induced migration of keratinocytes.

### 2.4. Gintonin-Induced Expression and Release of HB-EGF in HaCaT Cells

**Figure 5:**
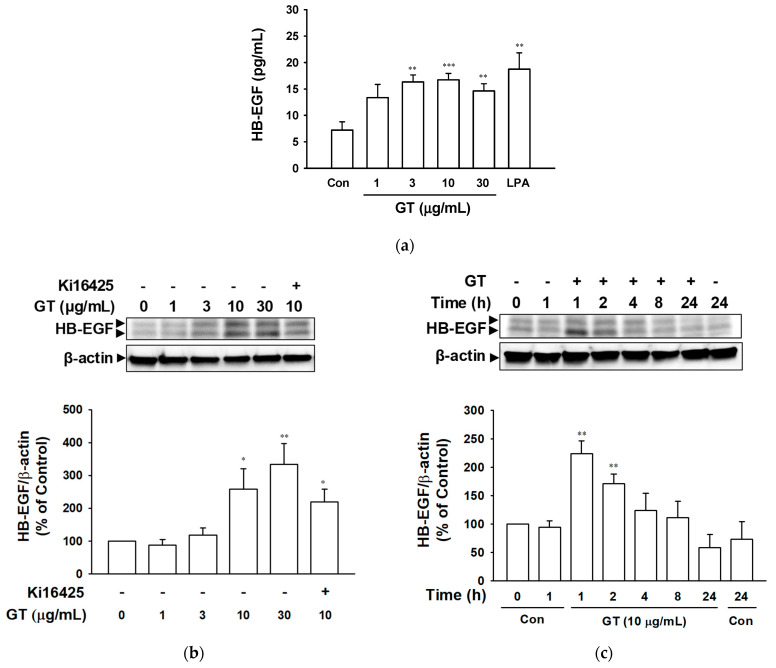
HB-EGF release and expression by gintonin in HaCaT cells. (**a**) Cells were incubated in serum-free medium containing gintonin (GT, 1–30 μg/mL) or lysophosphatidic acid (LPA, 10 μM) for 24 h and HB-EGF release in the conditioned medium was detected via ELISA. (**b**) Cells were incubated in serum-free medium containing GT (1–30 μg/mL) for 60 min in the presence or absence of LPA receptor inhibitor Ki16425 (10 μM). Then, HB-EGF expression in the cell lysate was detected via immunoblotting. (**c**) Cells were incubated with GT (10 μg/mL) for the indicated times and then cell lysates were immunoblotted for the detection of HB-EGF expression. Each protein level in untreated cells (Con) was considered to be 100%. Data are presented as means ± S.E.M. (*n* = 4); * *p* < 0.05; ** *p* < 0.01; *** *p* < 0.001 vs. untreated control cells. HB-EGF, heparin-binding epidermal growth factor-like growth factor.

HB-EGF is known as one of the potent ligands of EGF receptors [1,20,21,22]. Thus, we hypothesized that the delayed and sustained EGF receptor activation pattern induced by gintonin might be related to the gintonin-mediated release of other EGF receptor ligands, including HB-EGF. To determine the release and expression of HB-EGF in HaCaT cells exposed to gintonin, we treated HaCaT cells with gintonin and analyzed the release of HB-EGF in a conditioned medium after cell culture and the expression of HB-EGF in cells using an enzyme-linked immunosorbent assay (ELISA) and immunoblotting, respectively. Gintonin significantly increased HB-EGF release in the concentration range of 3 to 30 μg/mL for 24 h (Figure 5a). LPA (10 μM) also increased HB-EGF release. In addition, gintonin increased the expression of HB-EGF in cells in the concentration range of 3 to 30 μg/mL in a dose-dependent manner, which was significant at 10 and 30 μg/mL (Figure 5b). In HaCaT cells incubated with gintonin (10 μg/mL) for 1 to 24 h, the expression level of HB-EGF showed a significant increase after 1 and 2 h of incubation and then gradually decreased in a time-dependent manner until 24 h of incubation (Figure 5c).

### 2.5. The Relationship of EGF Receptor and Expression of HB-EGF in HaCaT Cells Exposed to Gintonin

**Figure 6:**
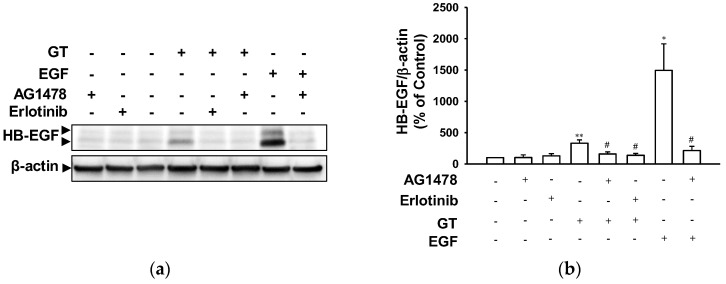
Effect of EGF receptor inhibitors on gintonin-induced HB-EGF expression in HaCaT cells. Cells were incubated with serum-free medium containing gintonin (GT, 10 μg/mL) or epidermal growth factor (EGF, 4 ng/mL) for 60 min in the presence or absence of EGF receptor inhibitor erlotinib (0.5 μM) or AG1478 (0.5 μM). Then, cell lysates were immunoblotted for the detection of HB-EGF expression. (**a**) Representative images of immunoblotting. (**b**) Statistical graph obtained from panel (**a**). Graph is expressed as a percentage of untreated control cells. Data are presented as means ± S.E.M. (*n* = 4); * *p* < 0.05; ** *p* < 0.01 vs. untreated control cells; ^#^ *p* < 0.05 vs. GT or EGF alone. HB-EGF, heparin-binding EGF-like growth factor.

To examine whether or not EGF receptor signaling is involved in the gintonin-induced expression of HB-EGF in HaCaT cells, we analyzed HB-EGF expression in HaCaT cells exposed to gintonin (10 μg/mL) via immunoblotting. The gintonin-induced increase in HB-EGF expression in HaCaT cells was blocked via treatment with the EGF receptor inhibitor erlotinib or AG1478 (Figure 6). These results indicate that the gintonin-enhanced expression of HB-EGF in HaCaT cells may have been due to the activation of the EGF receptor.

## 3. Discussion

In this study, we examined the EGF-like effects of gintonin. Gintonin appeared to activate the EGF receptor via the phosphorylation of the EGF receptor (Figure 1a). Delayed EGF receptor phosphorylation by gintonin was observed in comparison to direct EGF receptor phosphorylation by EGF, suggesting that gintonin-induced EGF receptor activation may not be accomplished directly by gintonin (Figure 1b). LPA receptor subtypes LPA1, 4–6 were highly expressed in HaCaT cells [29,30]. Gintonin acts as an LPA ligand as determined through much research on various cell types, including keratinocytes [25,26,27,28,29]. Pretreatment with the LPA 1/3 receptor inhibitor Ki16425 blocked the gintonin-induced proliferation and migration of HaCaT keratinocytes [29]. The LPA1/3 receptor inhibitor Ki16425 in this study completely blocked gintonin-induced EGF receptor phosphorylation. These results suggest that LPA receptor activation is linked to EGF receptor phosphorylation and activation.

Previously, it was reported that the stimulation of human keratinocyte HaCaT cells with thrombin or LPA enhanced the tyrosine phosphorylation of the EGF receptor [16,17]. Subsequent SHC tyrosine phosphorylation and complex formation with Grb2 and Src tyrosine phosphorylation are related to downstream Ras/mitogen-activated signaling [16,17], one of the main signaling pathways involved in cell proliferation. Muscarinic receptor activation by acetylcholine also transactivates the EGF receptor [17,31] and contributes to keratinocyte migration [1,32]. In this study, pretreatment with an EGF receptor inhibitor completely blocked gintonin-mediated ERK phosphorylation (Figure 2) and the proliferation of HaCaT cells (Figure 3). In addition, the gintonin-induced migration of keratinocytes was dramatically inhibited via treatment with EGF receptor inhibitors (Figure 4). Tyrosine kinase inhibitors, including erlotinib and AG1478, inhibit the EGF receptor by binding to their ATP pockets [33,34]. These results suggest that gintonin stimulates ERK phosphorylation via GPCR LPA receptor activation and EGF receptor transactivation, resulting in the proliferation and migration of HaCaT cells.

Various growth mediators can stimulate the growth of keratinocytes, which are the main components of the epidermis. In addition to EGF, fibroblast growth factors, interleukin-6, transforming growth factor-α (TGF-α), and HB-EGF have been reported as positive growth mediators in keratinocytes [1]. The most important growth factors are members of the EGF family, including TGF-α and HB-EGF, which are secreted by keratinocytes and are classified as autocrine growth factors. HB-EGF is an autocrine and paracrine keratinocyte growth factor that binds to EGF receptors to stimulate keratinocyte cell growth [20]. Hashimoto et al. reported that TGF-α or EGF induces HB-EGF mRNA expression, whereas HB-EGF induces HB-EGF mRNA and TGF-α mRNA expression in human keratinocytes [20]. HB-EGF and EGF also induce TGF-α- protein production [20]. Thus, HB-EGF, TGF-α, and EGF bind to and mutually amplify EGF receptors [20]. On the other hand, it has been reported that cellular stress conditions induced via cholesterol depletion, lipid raft disruption, and ATP release induce HB-EGF expression and activate the EGF receptor and ERK signaling [24].

In this study, gintonin treatment stimulated both the expression of HB-EGF and the release of soluble HB-EGF in HaCaT cells (Figure 5). Moreover, the gintonin-induced expression of HB-EGF was reduced by an EGF receptor inhibitor and not by a LPA1/3 receptor inhibitor, suggesting that it is likely independent of LPA1/3 receptor activation, but is closely related to EGF receptor activation. Furthermore, EGF receptor activation resulted in an increased expression and release of HB-EGF (Figure 5a and Figure 6). It is likely that soluble HB-EGF binds to the EGF receptor and activates it, acting as an autocrine growth factor in keratinocytes. As mentioned above, EGF, TGF-α, and HB-EGF may also act as mutual amplifiers. However, it was previously demonstrated that HB-EGF also could be induced even by cellular stress and ATP release. In mouse astrocytes, gintonin treatment increased ATP release in a concentration- and time-dependent manner, and gintonin-mediated ATP release in astrocytes was partially, but not completely, attenuated via treatment with the LPA receptor inhibitor Ki16425 [35]. These results indicated that LPA receptor-independent signaling pathways, including enhanced ATP release, are involved in gintonin-induced HB-EGF expression.

In a previous report, we suggested that gintonin induces the proliferation and migration of keratinocytes through LPA receptor activation and VEGF release during wound healing [29]. The major downstream signaling pathways of LPA and EGF receptor activation include the PLC/Ca^2+^, PLC/PKC, Ras/MAPK (MEK/ERK), and PI3K/AKT pathways [14,15]. Downstream signaling pathways of VEGF activation include the PI3K/AKT pathways [29,36]. HB-EGF can also induce VEGF production depending on PI3K and MAPK signaling [36]. Taken together, gintonin may activate the LPA, EGF, and VEGF receptors and seems to share these downstream pathways. Additionally, gintonin may induce the expression and release of HB-EGF in a manner dependent on or independent of LPA receptor activation, contributing to the proliferation and migration of keratinocytes and skin wound repair.

**Figure 7:**
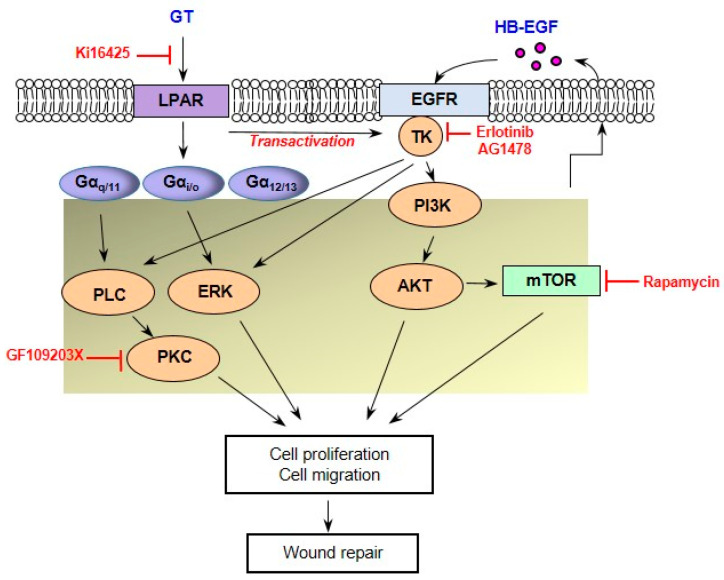
Possible signaling pathways involved in the gintonin-induced proliferation and migration of keratinocytes. Gintonin induces LPA receptor activation and EGF receptor transactivation in keratinocytes. These responses may activate PLC/PKC, ERK and PI3K/AKT/mTOR through G-proteins, leading to keratinocyte proliferation and migration. In addition, HB-EGF release also may activate the autocrine EGF receptor in keratinocytes. These signaling pathways may contribute to keratinocyte migration and proliferation, which are linked to skin wound repair. GT, gintonin; LPAR, lysophosphatidic acid receptor; EGFR, epidermal growth factor receptor; ERK, extracellular signal-regulated kinase; PLC, phospholipase C; PKC, protein kinase C; PI3K, phosphoinositide 3-kinases; AKT, protein kinase B; mTOR, The mammalian target of rapamycin; TK, tyrosine kinase.

In conclusion, the present study demonstrated that treatment with EGF and LPA receptor inhibitors markedly blocked gintonin-enhanced EGF receptor phosphorylation, cell proliferation, and migration. The treatment of HaCaT cells with gintonin also increased the expression and release of HB-EGF, an autocrine EGF receptor activator. These results indicate that HB-EGF may contribute to the gintonin-induced increase in keratinocyte proliferation and migration and that gintonin may induce the proliferation and migration of keratinocytes by activating LPA receptor–EGF receptor transactivation-mediated downstream signaling pathways (Figure 7). Therefore, gintonin may be a beneficial material that can be useful in the development of pharmaceuticals or cosmetics for skin wound healing.

## 4. Materials and Methods

### 4.1. Materials

Gintonin was obtained from *Panax ginseng* as in previous reports [25,37]. 1-Oleoyl-2-hydroxy-sn-glycero-3-phosphate (LPA C18:1) was purchased from Avanti Polar Lipids (Alabaster, AL, USA). Anti-phospho-EGF receptor(EGFR), anti-EGFR, anti-phospho-ERK1/2, and anti-ERK1/2 antibodies were purchased from Cell Signaling Technology, Inc. (Danvers, MA, USA). Anti-β–actin-horse radish peroxidase-conjugated antibodies were purchased from Abcam (Cambridge, MA, USA). Goat anti-rabbit IgG antibodies were purchased from GeneTex (Irvine, CA, USA). Goat anti-HB-EGF antibody and an ELISA kit for HB-EGF detection were purchased from R&D Systems (Minneapolis, MN, USA). Ki16425 was purchased from Cayman Chemical Co. (Ann Arbor, MI, USA). Dulbecco’s modified Eagle’s medium (DMEM) (low-glucose) was purchased from Welgene Inc. (Gyeongsan-si, Gyeongsangbuk-do, Republic of Korea). All other materials and EGF were purchased from Thermo Fisher Scientific (Seoul, Republic of Korea).

### 4.2. Cell Culture

The human skin keratinocyte cell line HaCaT was provided by Prof. H. M. Lee (Hoseo University, Asan-si, Chungcheongbuk-do, Republic of Korea) [29]. HaCaT cells were cultured in DMEM containing fetal bovine serum (FBS) (10% (*v*/*v*)), penicillin (100 units/mL), and streptomycin (100 μg/mL).

### 4.3. Cell Proliferation Assay

Cell proliferation was analyzed with a water-soluble tetrazolium formazan (WST)-based assay with EZ-Cytox (Dogen, Seoul, Republic of Korea), in accordance with the manufacturer’s instructions. Briefly, cells were plated into 96-well plates at a density of 5 × 10^3^ cells per well and incubated for 48 h. The cells were serum-starved for 4 h, pretreated with the inhibitors for 30 min, and then treated with gintonin, LPA, or EGF for 24 h [29]. After replacing the culture medium with fresh serum-free medium without phenol red, the cells were then treated with the EZ-Cytox solution for 2 h. Absorbance was measured at 450 nm using a Spectra Max 190 plate reader (Molecular Devices, Sunnyvale, CA, USA).

### 4.4. Migration Assay 

HaCaT cell migration was assessed using modified Boyden chambers (Neuro Probe, Gaithersburg, MD, USA) as in previous reports [25,26,29]. Cells (5 × 10^4^ cells/well) were loaded into the lower chambers and assembled in the presence or absence of inhibitors. The chamber was reversed and incubated at 37 °C for 1 h. The chamber was placed in an upright position. Gintonin, LPA, or EGF in serum-free DMEM was added to the upper chamber. Migrated cells were fixed and stained with Diff Quik (Sysmex, Kobe, Japan), photographed using a dark field microscope (Eclipse 80i; Nikon, Tokyo, Japan) at ×200, and counted.

### 4.5. HB-EGF ELISA

HB-EGF levels were detected as previously reported [24], with some modifications. Briefly, HaCaT cells were incubated with serum-free DMEM at 37 °C for 4 h and with DMEM in the presence or absence of different concentrations of gintonin for 24 h. In accordance with the manufacturer’s instructions, HB-EGF content in the culture medium supernatant (conditioned medium) was measured using an ELISA kit (R&D Systems, Minneapolis, MN, USA).

### 4.6. Immunoblotting

Phosphorylation levels of EGF receptor and ERK in HaCaT cell lysates were analyzed. Cells were lysed with a modified radioimmunoprecipitation assay (RIPA) buffer containing phosphatase and protease inhibitors. Each protein was separated via 4–20% gradient sodium dodecyl sulfate-polyacrylamide gel electrophoresis (SDS-PAGE) and transferred at 4 °C to polyvinylidene fluoride membranes. Membranes were incubated with rabbit anti-phospho-EGFR polyclonal antibody (1:1000), rabbit anti-phospho-ERK antibody (1:1000), and goat anti-rabbit IgG antibody conjugated to HRP. The membrane was stripped and re-probed with a rabbit anti-EGF receptor polyclonal antibody (1:1000) or rabbit anti-ERK antibody (1:1000). HB-EGF was detected using an HB-EGF antibody as previously described [24]. For the detection of loading controls for some experiments, mouse anti-β-actin monoclonal antibody conjugated to HRP (1:30,000) was also used. Images were visualized using Clarity Western ECL Substrate (Bio-Rad, Hercules, CA, USA) and an iBright CL1000 imaging system (Thermo Fisher Scientific).

### 4.7. Statistical Analysis

Data are expressed as the means ± standard error of the mean (S.E.Ms). The significance of differences between pairs of groups was determined using Student’s *t*-test for comparisons, and multiple comparisons were performed via one-way analysis of variance (ANOVA) using GraphPad Prism (version 5.0; GraphPad Software, Inc., La Jolla, CA, USA). *p* values less than 0.05 were considered to indicate significant differences.

## Data Availability

Data is contained within the article.
